# ANGPTL2/LILRB2 signaling promotes the propagation of lung cancer cells

**DOI:** 10.18632/oncotarget.4217

**Published:** 2015-05-20

**Authors:** Xiaoye Liu, Xiaoting Yu, Jingjing Xie, Mengna Zhan, Zhuo Yu, Li Xie, Hongxiang Zeng, Feifei Zhang, Guoqiang Chen, Xianghua Yi, Junke Zheng

**Affiliations:** ^1^ Institute of Health Sciences, Shanghai Institute for Biological Sciences, University of Chinese Academy of Science, Chinese Academy of Sciences and Shanghai Jiao Tong University School of Medicine, Shanghai, China; ^2^ Hongqiao International Institute of Medicine, Shanghai Tongren Hospital, Faculty of Basic Medicine, Shanghai Jiao Tong University School of Medicine, Shanghai, China; ^3^ Key Laboratory of Cell Differentiation and Apoptosis of Chinese Ministry of Education, Shanghai Jiao Tong University School of Medicine, Shanghai, China; ^4^ Department of Pathology, Tongji Hospital, Tongji University School of Medicine, Shanghai, China; ^5^ Bingzhou Medical University, Taishan Scholar Program, Yantai, China; ^6^ Ruijin Hospital, Shanghai Jiao Tong University School of Medicine, Shanghai, China

**Keywords:** ANGPTL2/LILRB2 signaling, lung cancer, metastasis, CaMK1

## Abstract

Immune inhibitory receptors expressed on various types of immune cells deliver inhibitory signals that maintain the homeostasis of the immune system. Recently we demonstrated that leukocyte immunoglobulin-like receptor subfamily B member 2 (LILRB2) and its murine homolog, paired immunoglobulin-like receptor B (PIRB), are expressed on hematopoietic stem cells and acute myeloid leukemia stem cells and function in maintenance of stemness. Herein, we determined that both LILRB2 and its soluble ligand ANGPTL2 are highly expressed in non-small cell lung cancer (NSCLC) samples, and levels are adversely related to patient prognosis. Inhibition of LILRB2 expression in NSCLC cell lines, such as A549 cells, resulted in a dramatic decrease in proliferation, colony formation, and migration. Mechanistic analyses indicated that ANGPTL2 binds LILRB2 to support the growth of lung cancer cells and that the SHP2/CaMK1/CREB axis controls the proliferation of lung cancer cell lines. Our results suggest that signaling involving ANGPTL2 and LILRB2 is important for lung cancer development and represents a novel target for treatment of this type of cancer.

## INTRODUCTION

According to data from American Cancer Society (2000-2014), lung cancer is the second most common type of tumor and the leading cause of cancer deaths worldwide [1]. In the past several decades, the occurrence of lung cancer has dramatically increased. Based on histology, there are two major types of lung cancer: non-small cell lung cancer (NSCLC) and small cell lung cancer (SCLC). Around 85% of the lung cancer patients have NSCLC; NSCLC is further divided squamous cell carcinoma, adenocarcinoma, and large cell lung cancer subtypes [2]. About 40% of lung cancers are adenocarcinomas, and the incidence of this subtype is tightly correlated with smoking [3, 4]. Mutations or abnormal expression of a number of molecules, including EGFR, KRAS, TITF1, B7-H1, and epigenetic regulators, are correlated with initiation and development of lung cancers [5-9]. EGFR inhibitors, angiogenesis inhibitors, and monoclonal antibodies have been analyzed in clinic trials with promising results in some subtypes of lung cancer [10, 11]. The mechanisms underlying lung cancer initiation, progression, and metastasis remain largely unknown and patients will benefit from a better understanding of this disease.

ANGPTLs are a family of secreted glycoproteins that have the same domain structure as angiopoietins; members of both families have an N-terminal coiled-coil domain and a C-terminal fibrinogen-like domain [12]. Unlike angiopoietin 1 and 2, which bind tyrosine kinase receptors Tie1 or Tie2, ANGPTLs do not bind Tie1 or Tie2 [13]. ANGPTLs are widely expressed in many tissues including liver, vascular system and hematopoietic system, and play important roles in inflammation, lipid metabolism, and angiogenesis [14, 15, 12, 16-19]. ANGPTLs are also involved in the regulation of cell proliferation and metastasis in many types of solid tumors. For example, ANGPTL1 can suppress SLUG to inhibit cancer cell motility [20], and ANGPTL3 plays an important role in cancer growth and invasion [9]. ANGPTL4 is highly expressed in many tumors and promotes tumor growth and decreases apoptosis [21]. TGF-β primes breast tumors for lung metastasis seeding through ANGPTL4 [22]. ANGPTL2 is not only an important facilitator of inflammatory carcinogenesis but also may represent a potential marker of breast cancer metastasis [23, 24]. The functions of ANGPTLs in lung cancer remain largely undefined.

Several ANGPTLs (ANGPTL 1, ANGPTL2, ANGPTL3, ANGPTL5, and ANGPTL7) can stimulate the activities of both human and mouse hematopoietic stem cells [25-27]. Recently, we showed that LILRB2 is a receptor for several ANGPTLs including ANGPTL1, ANGPTL2, and ANGPTL5 [19]. Interestingly, LILRB2 and its mouse homolog, PIRB, are highly expressed on hematopoietic stem cells (HSCs) and leukemic stem cells (LSCs) and are critical to maintenance of stemness. LILRB2 belongs to the LILR family. Six members of LILRA (LILRA1-LILRA6) and five members of LILRB (LILRB1-LILRB5) have been identified. Unlike members of the LILRA family, LILRB family receptors contain ITIM motifs that can recruit phosphatases, like SHP1 or SHP2, to mediate downstream inhibitory signaling and negatively regulate immune activation [28, 29]. LILRBs are expressed on many solid tumors. LILRB1 and LILRB4 are expressed in human gastric cancer cells and may enhance tumor growth [30]. LILRB2 is mainly expressed on myeloid cells and B cells and acts to suppress the immune response; it is also expressed on NSCLC cells [31]. We recently demonstrated that calcium/calmodulin-dependent protein kinase (CaMK) signaling is increased upon ANGPTL stimulation in hematopoietic cells, suggesting that CaMK is a mediator of the ANGPTL/LILRB2 signaling [19]. No other downstream targets of ANGPTL/LILRB2 have been identified.

In this report, we demonstrate that ANGPTL2/LILRB2 signaling regulates the proliferation and migration of A549 cells, an NSCLC line. Inhibition of LILRB2 expression significantly decreased the proliferative ability of A549 cells, whereas the presence of ANGPTL2 dramatically enhanced the growth of A549 cells. Our data indicate that the LILRB2/SHP2/CAMK1/CREB signaling axis plays a critical role in the survival and migration of A549 cells. This finding suggests that targeting LILRB2 or factors downstream will effectively treat lung cancer.

## RESULTS

### ANGPTL2 and LILRB2 are highly expressed on human NSCLC cell lines and human lung cancer samples and expression levels reversely correlate with the survival of lung cancer patients

**Figure 1:**
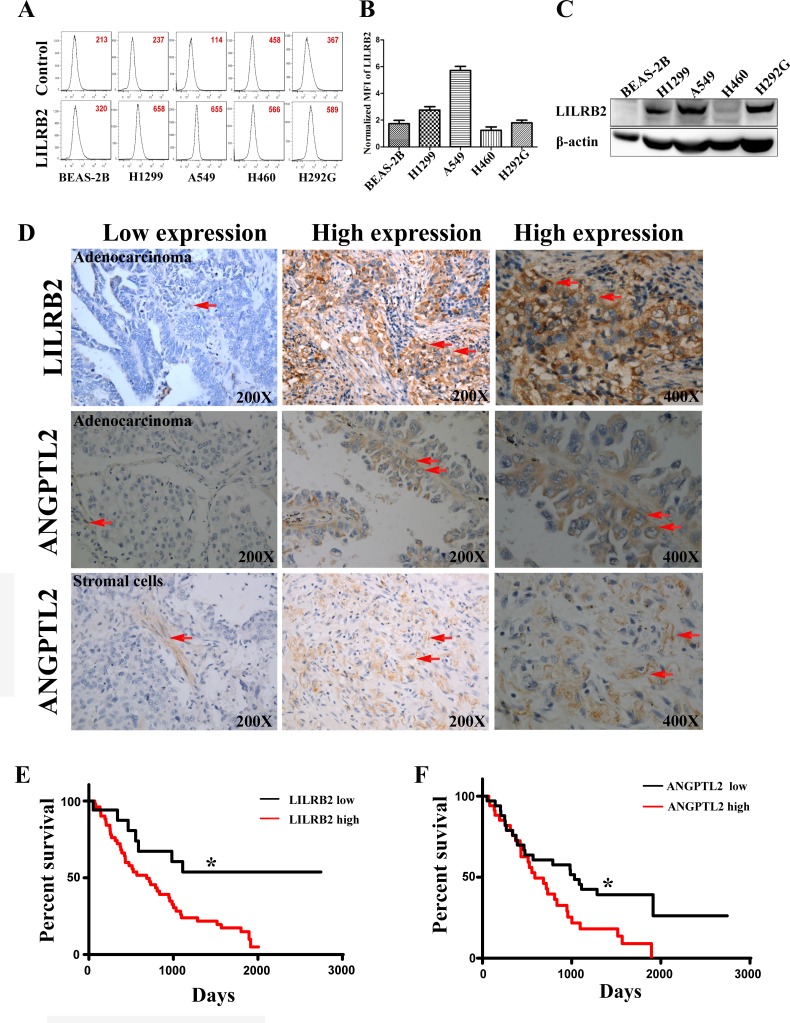
LILRB2 is highly expressed on human cancer cell lines and in primary tumor samples **A.** Representative flow cytometric analysis of LILRB2 expression in H1299, A549, H460, and H292G immortalized NSCLC cells. Lung epithelial cells, BEAS-2B, served as control cells. Mean fluorescence intensity (MFI) is indicated. **B.** MFI of LILRB2 expression in indicated lung cancer cell lines. Data plotted are means of three replicates. **C.** Western blotting analysis of LILRB2 expression in BEAS-2B, H1299, A549, H460, and H292G cells. **D.** LILRB2 expression (top panel, arrows) and ANGPTL2 expression in adenocarcinoma and stromal cells (middle and bottom panel, arrows) in representative samples from NSCLC patients examined by immunohistochemical staining. **E.** Survival curves of NSCLC patients in the LILRB2 low (*n* = 17) and LILRB2 high (*n* = 51) groups determined by Kaplan-Meier analysis (*n* = 68; Long-rank test). **G.** Survival curves of NSCLC patients in the ANGPTL2 low (*n* = 28) and ANGPTL2 high (*n* = 40) groups as determined by Kaplan-Meier analysis (*n* = 68). *P < 0.05, log-rank test.

To examine the expression of LILRB2 in human lung cancer cell lines, we first evaluated LILRB2 expression on several NSCLC cell lines, including H1299, A549, H460, and H292G cells, by flow cytometry. H1299 and A549 cells highly expressed LILRB2 compared to the other cancer cell lines evaluated and to normal lung epithelial cells, BEAS-2B cells (Figure 1A-1B). We confirmed that H1299, A549, and H292G cells had much higher levels of expression of LILRB2 protein by western blotting (Figure 1C).

We further examined the expression of LILRB2 in primary tissues collected from lung cancer patients. A total of 77 samples, including 68 NSCLC specimens, were collected at Shanghai Tongji Hospital from 1998 to 2008 and were evaluated by immunohistochemical staining for LILRB2 (sTable 1). Among the NSCLC samples, 35 were adenocarinomas and 33 were squamous cell carcinomas. LILRB2 was expressed in 75.0% (51 out of 68) of NSCLC samples (Figure 1D, top panel). In samples that expressed LILRB2, usually around 70% of cells were LILRB2^+^ (Figure 1D, top panel). However, none of the normal lung tissue cells expressed LILRB2 (SFigure 1A). Intriguingly, LILRB2 was expressed in both adenocarcinoma (Figure 1D, top panel) and in squamous cell carcinoma samples (SFigure 1B). We also found that some stromal cells were positive for the LILRB2 (SFigure 1B-1C), which indicated the tumor microenviroment might be involved in the cancer development.

As ANGPTLl2 is a high affinity ligand for LILRB2, we hypothesized these tissues would also express ANGPTL2. As shown in Figure 1D, ANGPTL2 was expressed in lung cancer cells (middle panel; around 68% of cells in a typical positive sample expressed ANGPTL2) and in stromal cells (bottom panel, around 75% of were ANGPTL2^+^ cells). In 58.8% (40 out of 68) of the NSCLC tissue samples, ANGPTL2 expression was upregulated compared to normal paratumor cells (SFigure 1D). Moreover, ANGPTL2 also could be detected in several NSCLC cell lines, including H1299, A549, H460, and H292G cells by western blotting, but not normal in normal control cells (SFigure 1E). Importantly, levels of both LILRB2 and ANGPTL2 negatively correlated with overall survival of NSCLC patients (Figure 1E-1F). Our results suggest that the autocrine or paracrine signaling through ANGPTL2/LILRB2 is involved in the development of NSCLC.

### LILRB2 promotes the proliferation of A549 cells

**Figure 2:**
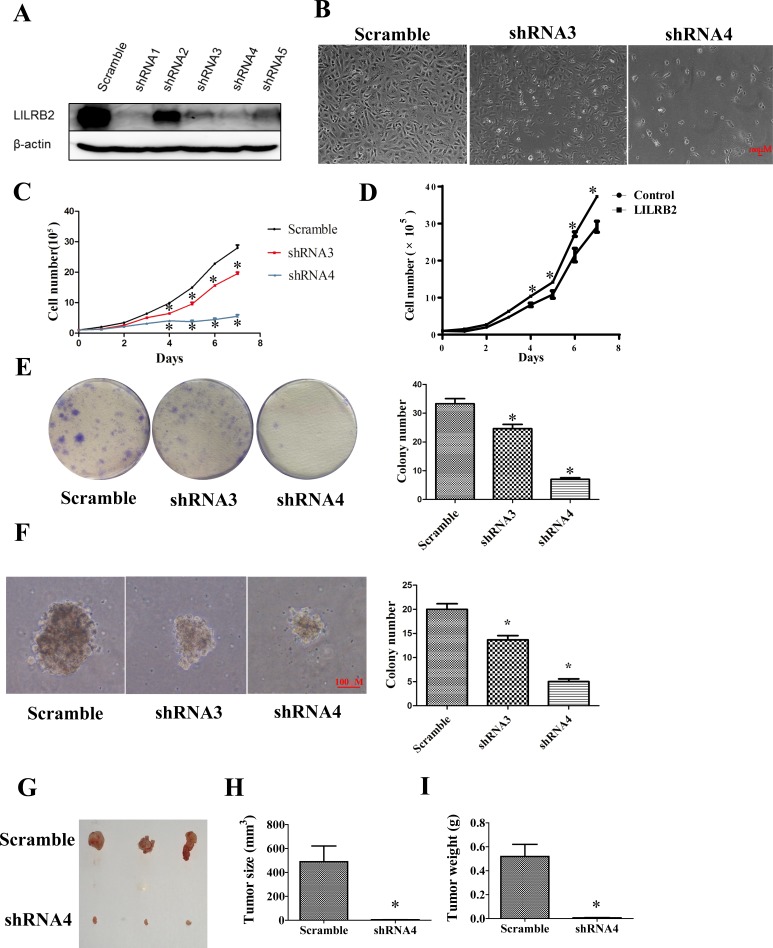
LILRB2 promotes the proliferation of A549 cells **A.** Western blotting analysis of A549 cells knockdowned with indicated shRNAs. **B.** Representative images of A549 cells 6 days after transfection with shRNA3 and shRNA4 targeting LILRB2 and with control shRNA (scramble). **C.** A549 cell numbers at indicated days after transfection with LILRB2-targeting shRNAs or control. Experiments were performed in triplicate. **D.** A549 cell numbers at indicated days after transfection with an LILRB2 overexpression vector or control vector. Experiments were performed in triplicate. **E.** Representative images of plates used in colony forming assays (left) and quantification of three replicates (right). A549 cells were knockdowned with shRNA3 and shRNA4 or control shRNA (scramble). **F.** Representative images of soft agar assay (left) and quantification of data (right). A549 cells were knockdowned with shRNA3 and shRNA4 or control shRNA (scramble). **G.**-**I.** NOD-SCID mice were inoculated with A549 cells that had been knockdowned with shRNA4 or with a scrambled control shRNA (*n* = 8). **G.** Photographs of representative tumors. **H.** Average tumor sizes and **I.** average tumor weights of one representative experiment with four animals. *P < 0.05.

Since A549 cells had the highest expression level of LILRB2 of the cultured cells evaluated, further experiments were performed in A549 cells. To explore the role of ANGPTL2/LILRB2 signaling in NSCLC, we inhibited LILRB2 expression in A549 cells using shRNAs (sTable 2). To examine the efficiency of the designed shRNAs, we co-transfected CMV-LILRB2 and each of five shRNAs into 293T cells and evaluated the expression of LILRB2 by western blotting 72 h after transfection. As shown in Figure 2A, shRNAs 1, 3, 4, and 5, efficiently inhibited LILRB2 expression, and this was further confirmed by flow cytometry (SFigure 2). In subsequent experiments, LILRB2 expression was inhibited in A549 cells by transfection with shRNA3 or shRNA4. Transfection with either of these shRNAs resulted in a dramatic decrease in proliferation as well as visible cell death (Figure 2B). Cell growth was much slower three days after transfection with LILRB2 shRNAs and was more apparent after seven days (Figure 2C); this may have resulted from increased apoptosis or disruption of the cell cycle. When LILRB2 was overexpressed in A549 cells, there was a dramatic increase in cell growth (Figure 2D). To further confirm the effect of LILRB2 in A549 cells, a colony forming assay was performed to investigate the changes in propagation ability. There were 24 ± 2 and 8 ± 1 colonies when cells were treated with shRNA3 and shRNA4, respectively, significantly fewer than the 34 ± 3 when cells were treated with a scrambled control shRNA (Figure 2E). A soft agar assay showed that the colony size was dramatically reduced after inhibition of LILRB2 expression. Colony numbers were decreased to 65 ± 1.5% and 25 ± 1.0% of the control level by shRNA3 and shRNA4, respectively (Figure 2F). Most strikingly, *in vivo* engraftment experiments clearly revealed that the tumor forming ability of A549 cells was almost totally abolished by knockdown of LILRB2 with shRNA4; tumor sizes and weights were much smaller than those in mice given cells knockdowned with the scramble control (Figure 2G-2I). Together, our data provide strong evidence LILRB2 supports the proliferation of solid cancer cells *in vitro* and *in vivo*.

### LILRB2 enhances the migration of A549 cells

**Figure 3:**
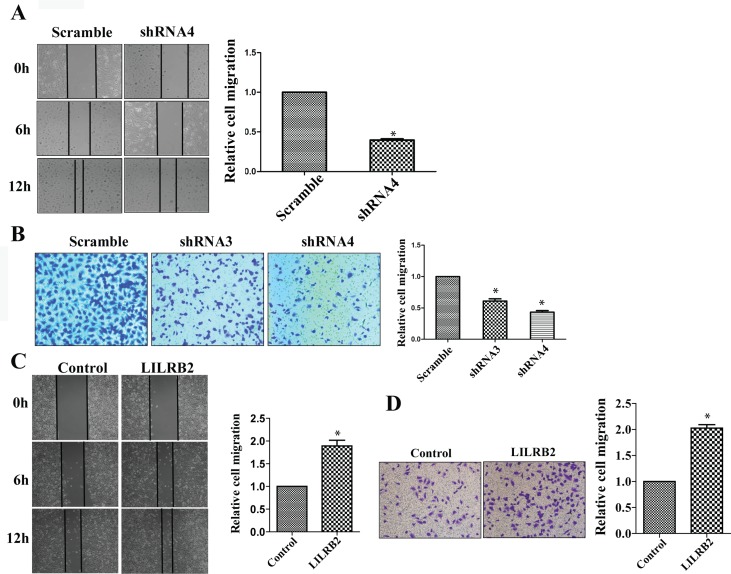
LILRB2 enhances the migration of A549 cells **A.** Representative images of wound healing assay of A549 cells knockdwoned with shRNA4 or scrambled control shRNA at indicated times (left). Quantification of data from three replicates (right). **B.** Representative images of transwell analyses performed to evaluate the migration ability of A549 cells after knockdown with shRNA3 and shRNA4 or with control shRNA (left). Quantification of data from three replicates (right). **C.** Representative images of wound healing assay for A549 transfected with a vector designed to overexpress of LILRB2 or with a control vector (left). Quantification of data from three replicates (right). **D.** Representative images of transwell analyses to evaluate the migration ability of A549 cells transfected with a vector designed to overexpress of LILRB2 or with a control vector (left). Quantification of data from three replicates (right). *P < 0.05.

ANGPTLs play a significant role in the migration of endothelial cells as well as metastasis of many solid tumors [24, 22, 32]. To investigate the possible role of ANGPTL/LILRB2 in invasion or migration of NSCLCs, we determined the migration ability of A549 cells after knockdown with LILRB2 shRNA. LILRB2-deficient A549 cells migrated more slowly (40 ± 1.5%) than cells knockdowned with a control shRNA in a wound healing assay (Figure 3A). We further confirmed the role of LILRB2 in migration using a transwell assay. Inhibition of expression of LILRB2 with either shRNA3 or shRNA4 led to a dramatic decrease in migration (44 ± 2.5% of control cells, Figure 3B). Overexpression of LILRB2 in A549 cells enhanced migration ability as measured in both the wound healing assay and the transwell assay (Figure 3C-3D).

### ANGPTL2 binds LILRB2 to promote proliferation of A549 cells

**Figure 4:**
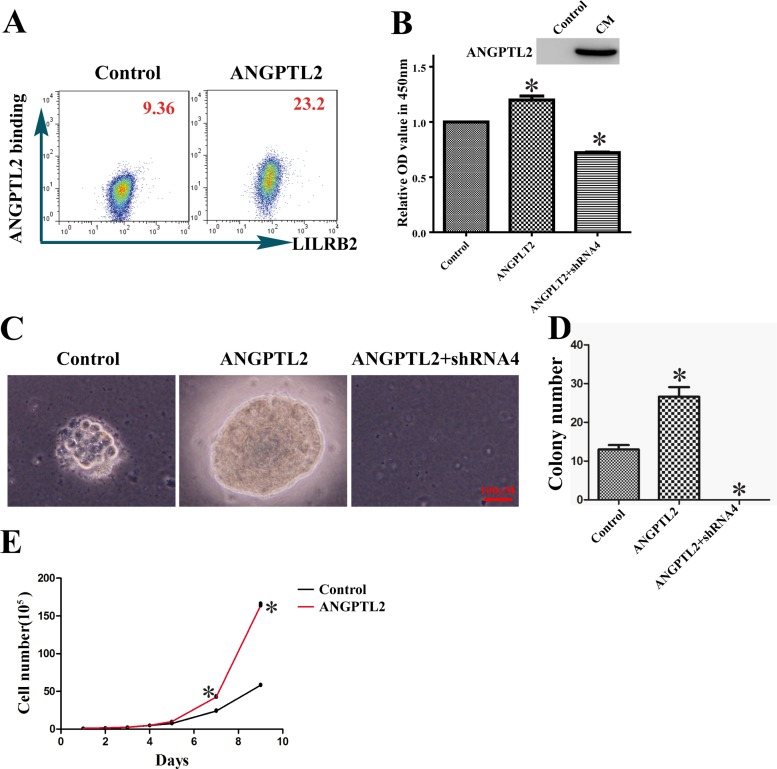
ANGPTL2 binds to LILRB2 and promotes proliferation of A549 cells **A.** The binding of LILRB2 to ANGPTL2 was analyzed by flow cytometry. Mean fluorescence intensity is indicated. **B.** ANGPTL2 level in conditional medium (CM) was examined by western blotting. Cell numbers were compared in cells with or without ANGPTL2 in medium. A549 cells knockdowned with shRNA4 targeting LILRB2 was also treated with ANGPTL2 and compared to control cells. **C.** Representative colonies in soft agar assay of A549 cells with or without ANGPTL2 in medium. A549 cells knockdowned with shRNA4 targeting LILRB2 was treated with ANGPTL2 and served as another control. **D.** Quantification of colony number in soft agar assay from three replicates. **E.** A549 cell numbers at indicated number of days with the overexpression of ANGPTL2 compared to control cells. *P < 0.05.

ANGPTL2 is a soluble ligand, and we studied the function of ANGPTL2 by growing A549 cells in its presence. First, using flow cytometry we demonstrated that ANGPTL2 indeed bound A549 cells through surface LILRB2 (Figure 4A). Next, we cultured A549 cells with or without conditional medium containing ANGPTL2 and examined its stimulatory effect. As shown in Figure 4B, the ANGPTL2 level in conditional medium (CM) was detected by western blotting and the ANGPTL2-treated cells proliferated to a greater extent than untreated cells. In contrast, the LILRB2-deficient A549 cells had a significant decrease of proliferation ability as compared to control cells although ANGPTL2 conditional medium was added for the culture. We further confirmed that ANGPTL2 induces proliferation in a soft agar assay. Colony sizes and numbers were significantly increased compared to control cells when A549 cells were cultured with ANGPTL2-containing conditioned medium (Figure 4C-4D). Finally, we overexpressed ANGPTL2 in A549 cells to verify that ANGPTL2 facilitated the propagation of A549 cells. The expression of ANGPTL2 and binding to LILRB2 were confirmed by western blotting and flow cytometry analyses, respectively (SFigure 3). Consistently, A549 cells that overexpressed ANGPTL2 grew more rapidly than control cells (Figure 4E). There were no obvious differences in migration upon ANGPTL2 overexpression, which suggests that autocrine signaling has only a minor effect on migration ability (SFigure 4).

### SHP2/CaMK1/CREB signaling regulates proliferation of A549 cells

**Figure 5:**
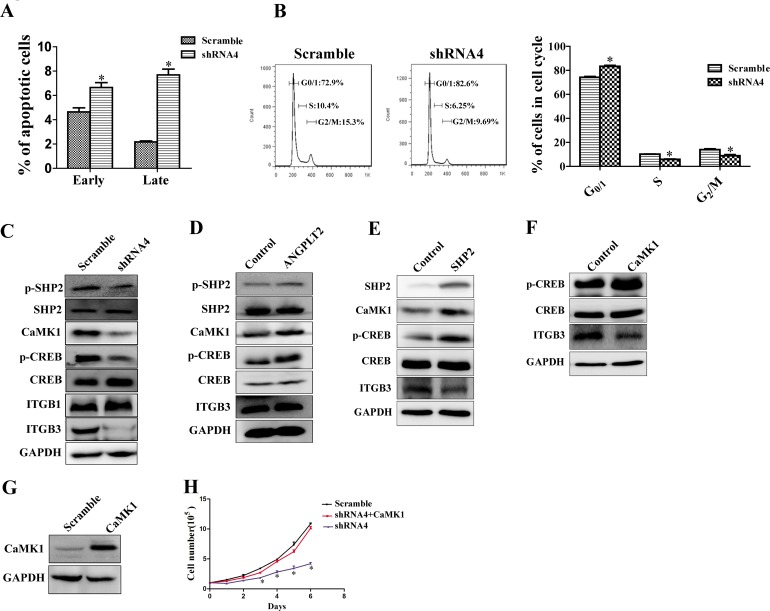
LILRB2/SHP2/CaMK1/CREB signaling regulates the proliferation of A549 cells **A.** Percent apoptosis was determined in A549 cells knockdowned with shRNA4 targeting LILRB2 or the scrambled control shRNA using Annexin V/PI staining. **B.** Stage of cell cycle was determined using PI staining in A549 cells treated with shRNA4 or the scrambled control shRNA (left, representative FACS plot for cell cycle; Right, quantitative analysis for cell cycle. **C.** SHP2/CaMK1/CREB or ITGB1/ITGB3 signaling was compared in A549 cells treated with shRNA4 or the scrambled control shRNA by western blotting analysis for the indicated proteins. **D.** SHP2/CaMK1/CREB or ITGB3 signaling was evaluated in A549 cells that overexpress ANGPTL2 and in control cells by western blotting analysis for the indicated proteins. **E.** CaMK1/CREB or ITGB3 signaling was evaluated in 293T cells that overexpress SHP2 and in control cells by western blotting analysis of the indicated proteins. **F.** CREB and ITGB3 signaling was evaluated in 293T cells that overexpress CaMK1 and in control cells by western blotting analysis. **G.** CaMK1 expression in CaMK1-overexpressed A549 cells detected by western blotting. **H.** Cell growth was rescued in LILRB2-knockdowned-A549 cells with overexpression of CaMK1. *P < 0.05.

To understand the mechanism by which ANGPTL2/LILRB2 controls tumor growth, we analyzed apoptosis and the cell cycle status of A549 cells knockdowned with shRNA targeting LILRB2 or with a control shRNA. There were more apoptotic cells and increased G0/G1 fraction in cells deficient in LILRB2 than in control cells (Figure 5A-5B). Both increased apoptosis and G1 arrest may contribute to dramatic slowdown of cell growth after LILRB2 knockdown in A549 cells. To identify downstream signaling networks activated by ANGPTL2 binding to LILRB2, we first examined SHP1 and SHP2 signaling. SHP1 was not detected. Phospho-SHP2 levels were significantly decreased in cells knockdowned with shRNA4 compared to those knockdowned with a control shRNA (Figure 5C). Previously, we reported that calcium/calmodulin-dependent protein kinases (CaMKs) play important roles in HSCs and LSCs. We thus examined the expression of CaMK family members in LILRB2-deficient cells. Only CaMK1 but not CaMK2 or 4 was detected, and CaMK1 levels were reduced in cells knockdowned with shRNA against LILRB2 (Figure 5C). Levels of phospho-CREB, which is the target of CaMK1, were also significantly decreased in LILRB2-deficient cells (Figure 5C). Consistently, levels of phospho-SHP2, CaMK1, and phospho-CREB were increased in A549 cells that overexpressed ANGPTL2 (Figure 5D). We then overexpressed SHP2 or CaMK1 in 293T cells. Consistently, overexpression of SHP2 resulted in robust increase of CaMK1 and phospho-CREB, and overexpression of CaMK1 induced a dramatic increase in phospho-CREB signal (Figure 5E-5F).

Next we introduced CaMK1 into A549 cells previously transfected with shRNA4 to inhibit LILRB2 expression. These cells, which were overexpressed CaMK1 (Figure 5G), cause tumor cell growth similar to levels of untreated A549 cells (Figure 5H) indicating that CaMK1 rescued the LILRB2 deficiency. To explore how LILRB2 regulates the migration of A549 cells, we examined levels of several relevant molecules including ITGB1 and ITGB3 by western blotting. ITGB3, but not ITGB1, was strikingly downregulated in LILRB2-deficient A549 cells (Figure 5C). However, ITGB3 was not upregulated by overexpression of ANGPTL2, SHP2, or CaMK1, which indicates that LILRB2 may directly interact with ITGB3 (Figure 5D-5F). To address this, we further performed a co-immunoprecipitation experiment and demonstrated that LILRB2 was directly associated with ITGB3 (SFigure 5). Although we demonstrated that ITGB3 was dramatically downregulated upon LILRB2 deletion in A549 cells, the underlying mechanism remains largely unknown. On the other side, in current system, since we use ANGPTL2 conditional medium to examine the effect of ANGPTL2/LILRB2 signaling in the control of cell migration, one potential problem is that we are not sure about the physiological concentration of ANGPTL2 to activate LILRB2, which may results in no significant effect of ANGPTL2 in regulating the migration of A549 cells by ANGPTL2/LILRB2 signaling. In summary, our results indicate that a signaling network involving SHP2, CaMK1, and CREB is regulated by ANGPTL2/LILRB2 in NSCLC cell lines.

## DISCUSSION

Most of studies of the immune inhibitory receptors such as those of the LILRB family have focused on their roles in the immune system [33, 28, 29]. Similarly, ANGPTLs have been demonstrated to play crucial roles in lipid metabolism and angiogenesis [34, 16, 35], and our previous study showed that LILRB2 inhibits differentiation and promotes self-renewal of HSCs and LSCs; however, the function of LILRB2 in cancer progression remains unclear. In this study, we provide evidence that both ANGPTL2 and LILRB2 are often expressed in lung cancer tissue and play important roles in the proliferation and survival of cancer cells. Inhibition of LILRB2 expression in A549 cells delayed cell growth and inhibited colony formation and migration. The effects of binding of ANGPTL2 to LILRB2 were mediated by the activation of downstream signaling through SHP2, CaMK1, and CREB.

Our data suggest that ANGPTL2 is a powerful driver of metastasis in lung cancer. ANGPTL4 promotes metastasis in several types of cancers including breast cancers and esophageal squamous cell carcinoma [36, 18], but the surface receptor for ANGPTL4 remains unknown. Although several ANGPTLs bind to LILRB2, ANGPTL4 does not. Further study will be necessary to determine whether other ANGPTLs, such as ANGPTL1, ANGPTL5, ANGPTL7, also enhance metastasis in lung cancer. Other LILRB members may have functions similar to those of LILRB2 demonstrated here. We suspect that it is possible that ANGPTLs/LILRBs form ligand-receptor clusters to regulate the initiation and development of lung cancer.

SHP1 and SHP2 are associated with LILRB family members and inhibit downstream signaling. We did not detect expression of SHP1 in A549 cells, but SHP2 enhanced CaMK signaling in our system. Four CaMK members have been identified; these proteins are expressed in many tissues and control cell proliferation, differentiation, and survival. Interestingly, CaMK2 and CaMK4 are highly expressed in hematopoietic cells and are activated upon ANGPTL2 stimulation. CaMK1 appears to be tightly controlled by LILRB2/SHP2 signaling in lung cancer cells. This suggests that CaMK members interact differently with ANGPTL2/LILRB2-mediated signaling in various systems. Our study also indicates that LILRB2 interacts with ITGB3 to regulate cell migration or metastasis.

Previously, we have showed ANGPTL2 promotes the self renewal and suppresses the differentiation of LSCs. In this study, although we don't have strong evidences to draw a conclusion about how ANGPTL2 regulates the stemness of lung cancer cells since we only use lung cancer cell lines to study its function, we speculate that ANGPTL2 may also play a role in both self renewal and inhibition of differentiation in lung cancer cells. Further studies on lung cancer stem cells *in vitro* or *in vivo* will provide more clear evidences for this. In summary, both ANGPTL2 and LILRB2 were highly expressed in NSCLC samples, and levels were adversely correlated with patient survival. ANGPTL2/LILRB2 binding provoked signals through the SHP2/CaMK/CREB axis as well as ITGB3 to facilitate pathways related to metastasis. Our results provide intriguing clues that ANGPTL2/LILRB2 triggers several signaling pathways to support the stemness (self-renewal and differentiation) and migration ability for both normal stem cells and tumor cells. Further effort will be necessary to identify other factors involved in ANGPTL2/LILRB2 signaling and the potential of inhibiting the functions of these factors for clinic applications.

## MATERIALS AND METHODS

### Cell culture

The human normal lung epithelial cell line BEAS-2B was obtained from the Chinese Academy of Sciences. A549 cells were obtained from the ATCC and other NSCLC cell lines, including H1299, H460, and H292G, were kindly provided by Prof. Jiong Deng (Shanghai Jiaotong University). BEAS-2B cells were cultured with Keratinocyte-SFM (Hyclone); A549 and H292G cells were cultured in Dulbecco's modified Eagle's medium (Hyclone) with 10% fetal bovine serum (FBS, Hyclone); H1299 and H460 cells were maintained in RPMI1640 (Hyclone) with 10% FBS. All cells were grown in a humidified atmosphere of 5% CO_2_ and 95% air.

### Patient samples and immunohistochemical staining

Lung cancer samples (including 68 NSCLC samples) were collected from 77 lung cancer patients who underwent surgery at Shanghai Tongji Hospital during the period from 1998 through 2008. Of these patients, 49 were men and 19 were women. All clinical information, including age, gender, smoking history, histological subtype, lymph node involvement, tumor node metastasis, and pathologic stage, is summarized in sTable 1. Patients were staged according to criteria described in the seventh edition of the UICC. All patients were until death or until November 1, 2014. All patients gave written informed consent. The Ethical Committee of Shanghai Tongji Hospital approved the tissue collection and studies with collected tumor tissues. Lung cancer tissues and adjacent normal tissues were fixed with 4% paraformaldehyde and embedded in paraffin for further sectioning. Immunohistochemical staining for LILRB2 and ANGPTL2 was performed with an antibody to LILRB2 (made in house) and with anti-ANGPTL2 (R&D Systems).

### Multiplicative quick score systems

Immunohistochemical stained slides were evaluated for Multiplicative Quick Score by two pathologists using the criteria mentioned in sTable 3 [34]. The score was calculated as the sum of the percentage of stained cells (1=0-4%, 2=5-19%, 3=20-39%, 4=40-59%, 5=60-79%, 6=80-100%) multiplied by a number (0-3) reflecting the intensity staining (0=negative, 1=weak, 2=moderate, 3=strong). If the score is less or equal to 2, it is defined as the low expression of LILRB2 or ANGPTL2. Otherwise, it represents the high expression of LILRB2 or ANGPTL2.

### Lentivirus construction and infection

The lentiviral vector Pll3.7 was used to express shRNAs designed to target LILRB2 (sequences listed in sTable 2). Lentiviral vectors PLVX-IRES-tdTomato and PLVX-IRES-zsgreen were used to construct CaMK1/LILRB2 and ANGPTL2/SHP2, respectively. Using calcium phosphate transfection method, lentivirus constructs together with the packaging plasmid pSPAX2 and pMD2G (4:3:1) were mixed and transfected into 293T cells. Supernatant containing lentivirus was harvested 48 h and 72 h later. After filtering through a 0.45-μm low protein binding-polysulfonic filter (Millipore), lentivirus were concentrated with Optima™L-100 XP ultracentrifuge (Beckman Coulter) and used for the following infection on A549 cells.

### Cell proliferation assay

A549 cells (100,000) treated with shRNA targeting LILRB2 or a scrambled shRNA were cultured in 6-well plates. Cell proliferation was evaluated by calculating the cell number at different time points or using the Cell Counting Kit-8 (CCK8 assay; Dojindo). For the CCK8 assay, cells were plated in a 96-well plate at 1-2×10^3^ cells/well. The absorbance was measured at 450 nm on day 3 and normalized to that of day 1.

### Transwell assay

Cell migration was determined using transwell insert chambers (8-mm pore size; Corning). A549 cells were harvested and resuspended in serum-free medium after infection. Approximately 3 × 10^4^ cells knockdowned with LILRB2-shRNA or scrambled control shRNA were added to the upper chamber and 10% FBS was added to lower chamber as the chemoattractant. After incubating at 37 °C for 24 h, migrated cells were fixed with 20% methanol, stained with 0.1% crystal violet (Invitrogen), and counted.

### Wound healing assay

When A549 cells reached 90% confluence after infection, migration ability was assessed by measuring the movement of cells into an area scraped with a sterile tip. Images were taken every 6 h (Nikon).

### Colony formation assay and soft agar assay

Approximately 1,000 A549 cells knockdowned with LILRB2-shRNA or a scrambled control shRNA were cultured in DMEM medium containing 10% FBS until colonies were visible (approximately 2 weeks). Colonies were fixed with cold methanol, stained with 0.1% crystal violet, and scored. For the soft agar assay, approximately 5 × 10^4^ cells were resuspended in DMEM medium containing 10% of FBS and mixed with 0.3% agar before adding to a 6-well plate coated with 0.6% agar. Cells were incubated at 37 °C until colonies were visible (approximately 2 weeks); all visible (diameter >100μm) colonies were counted.

### *In vivo* xenograft

shRNA4 was used to inhibit the expression of LILRB2 in A549 cells, and these cells or cells treated with a scrambled control shRNA were then used in the *in vivo* xenograft experiment. A549 cells (1 × 10^7^) were resuspended in 200 μl phosphate-buffered saline with 2% of FBS and injected subcutaneously to six-week-old NOD-SCID mice. Animal experiments were approved by the institution and conducted according to the Guideline for Animal Experiments. All the mice were sacrificed 4 weeks after injection. Tumor sizes and weights were determined.

### Western blotting and flow cytometry analysis

Whole cell lysates were electrophoresed on 10% sodium dodecyl sulfate polyacrylamide gels and transferred onto polyvinylidene difluoride membranes (Millipore). The membranes were incubated with primary antibodies overnight at 4°C followed by incubation with appropriate horseradish peroxidase-conjugated secondary antibodies. The following antibodies were used: anti-CREB, anti-Phospho-CREB (Ser133), anti-SHP-1, anti-Phospho-SHP1 (Tyr564), anti-SHP-2, anti-Phospho-SHP2 (Tyr580), anti-ITGB1, anti-ITGB3, p-CaMK2, CaMK2 (Cell Signaling Technology), anti-ANGPTL2 (R&D Systems), anti-CaMK1 (Abcam), anti-p-CaMK4 (Abmart), anti-CaMK4 (Genscript), anti-Flag (Sigma), anti-GAPDH, and anti-β-actin (Calbiochem). Anti-LILRB2-PE (eBioscience) and isotype control of IgG-PE were used to detect the expression of LILRB2 on the different lung cancer cell lines and analyzed by flow cytometry.

### Statistical analyses

All data are presented as the means ± SEM. Data were compared using two-tailed Student's *t*-test. Data were considered statistically significant at a value of *p* < 0.05. The survival between two groups was analyzed using a log-rank test.

## SUPPLEMENTARY MATERIAL FIGURES AND TABLES


