# Supplementary material for: ANGPTL2/LILRB2 signaling promotes the propagation of lung cancer cells
Source: Oncotarget. 2015 May 20;6(25):21004–15. doi: 10.18632/oncotarget.4217 (PMC4673246; doi:10.18632/oncotarget.4217)
Supplement: Supplementary file 1 [file oncotarget-06-21004-s001.pdf]

ANGPTL2/LILRB2 signaling promotes the propagation of lung cancer cells

Supplementary Material

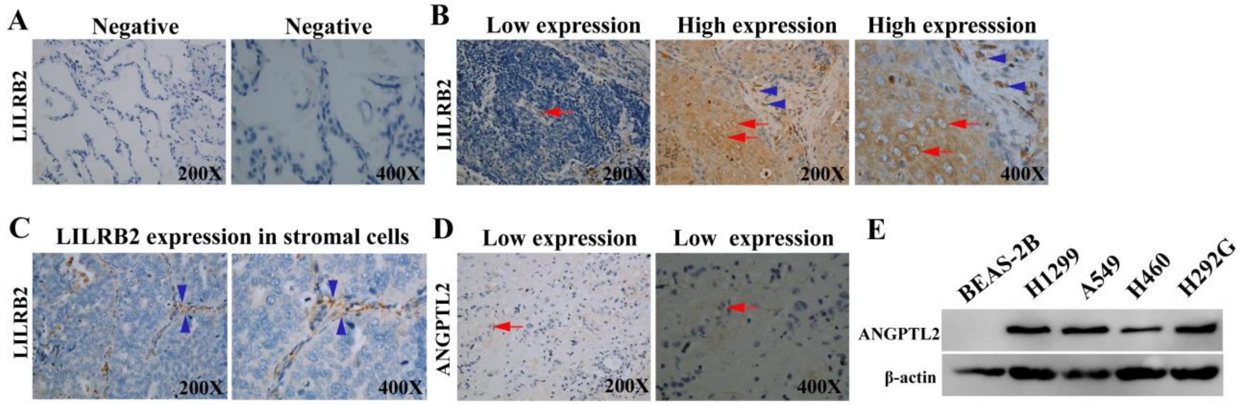

SFig 1: A) LILRB2 expression in paratumor tissue. B-C) LILRB2 expression in both lung squamous cell carcinoma (red, arrows) and tumor stromal cells (blue, arrowheads). D) ANGPTL2 expression in paratumor tissue (red, arrows). E) ANGPTL2 expression in NSCLC cell lines (H1299, A549, H460, and H292G) as well as normal control cells (BEAS-2B) evaluated by western blotting. Arrows and arrowheads indicate the LILRB2 or ANGPTL2 signals detected by immunohistochemical staining.

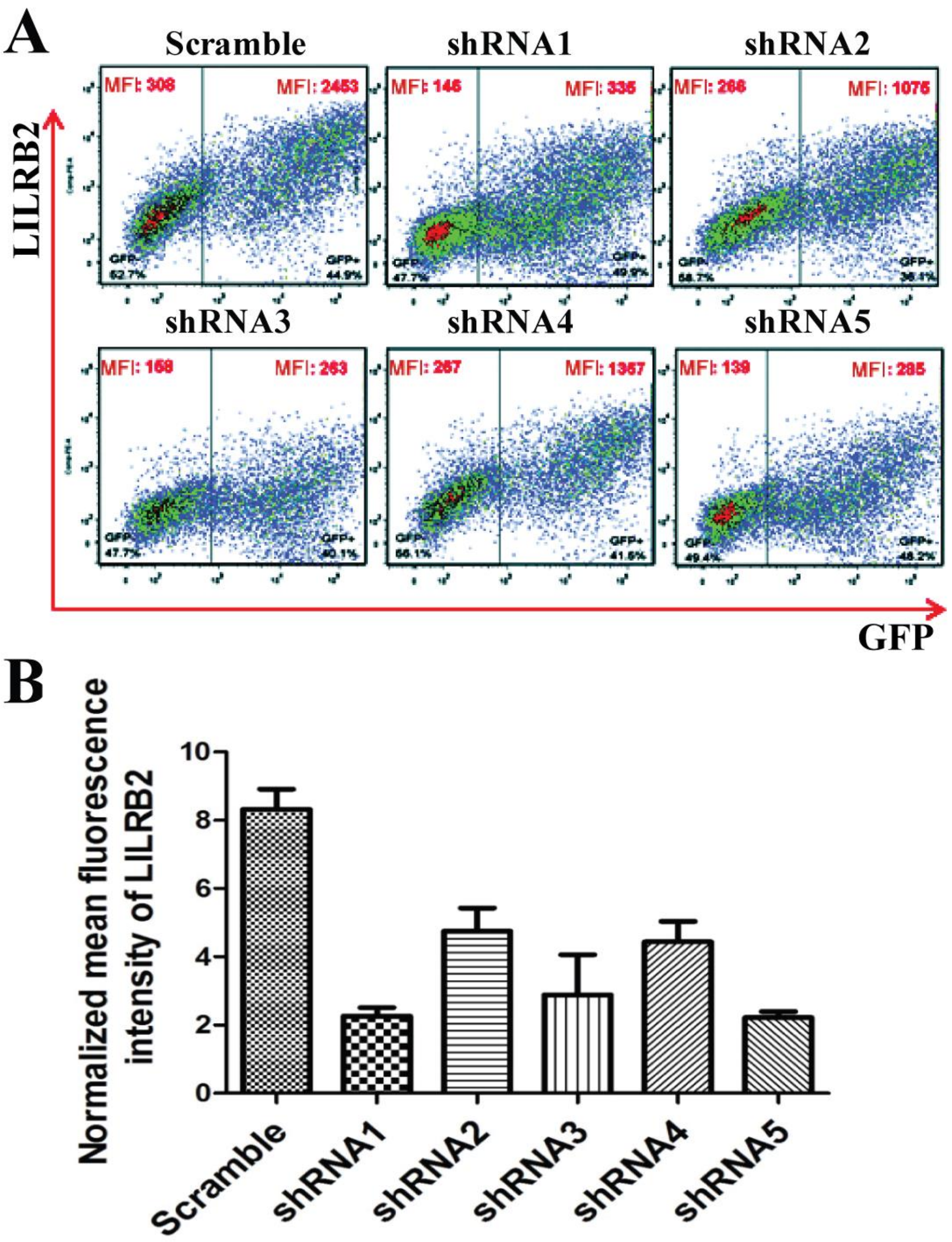

SFig 2: shRNAs targeting LILRB2 were transfected into A549 cells. A) Representative flow cytometry analyses of LILRB2 levels. B) Quantification of mean fluorescence intensity (MFI) in three replicates.

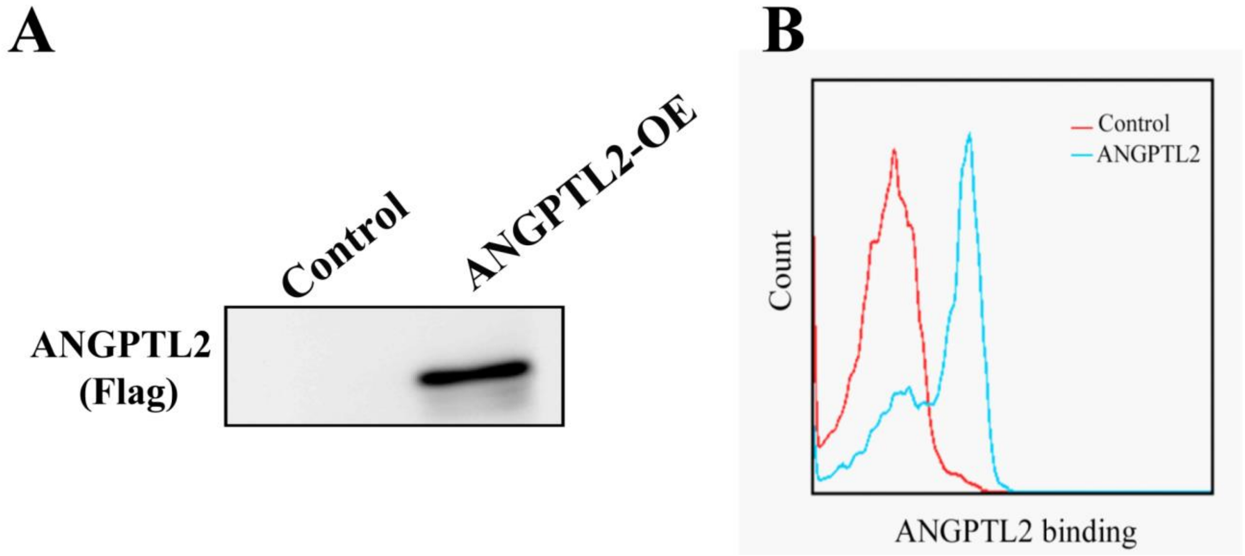

SFig 3: A) The expression level of ANGPTL2 in ANGPTL2-oeverexpressed A549 cells was examined by western blotting. B) The binding of secreted ANGPTL2 to LILRB2 on the surface of A549 cells was detected by flow cytometry.

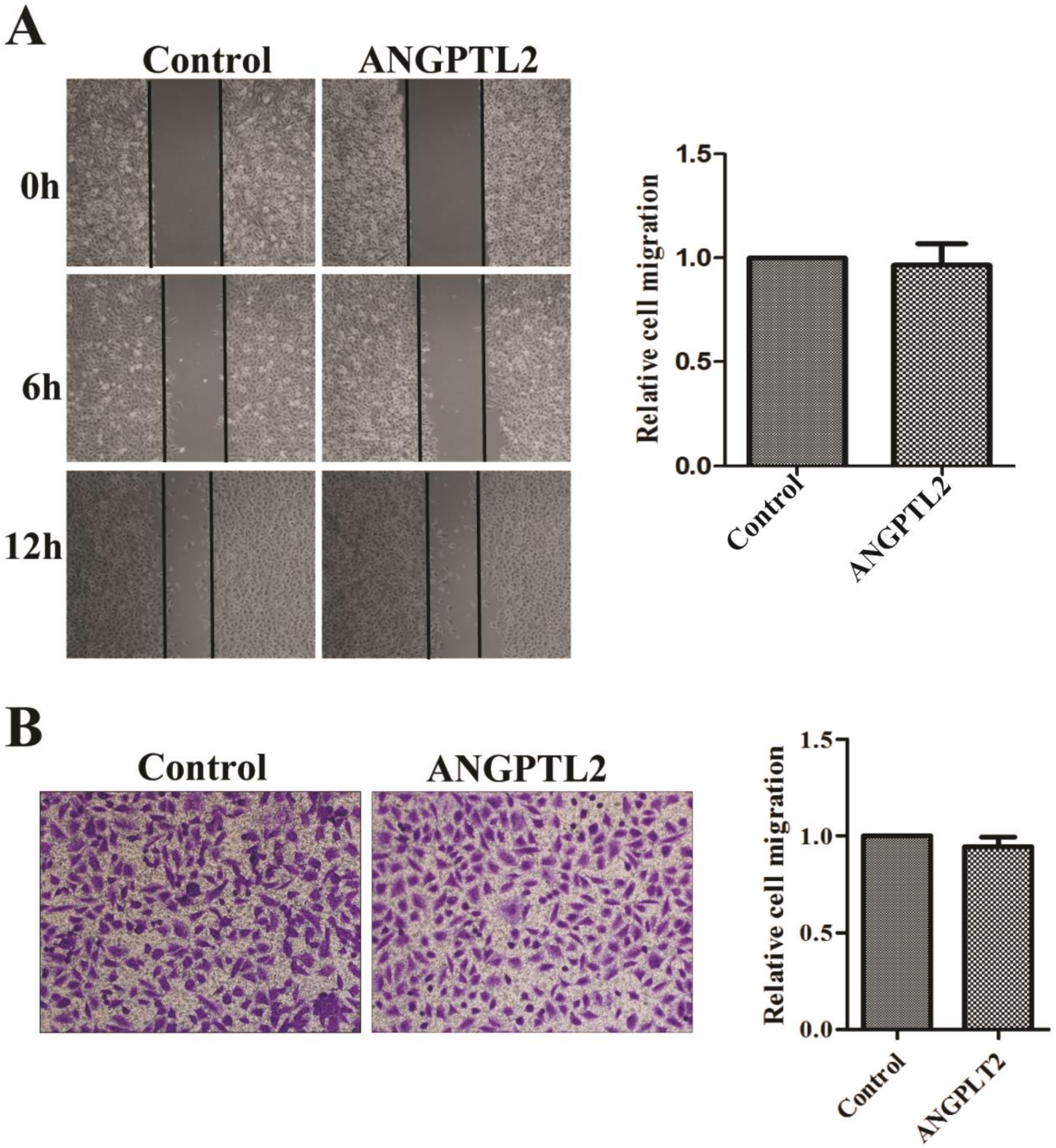

SFig 4: A) Wound healing assay comparing A549 cells that overexpress ANGPTL2 to those transfected with empty vector. B) Transwell analysis of A549 cells that overexpress ANGPTL2 compared to cells transfected with empty vector.

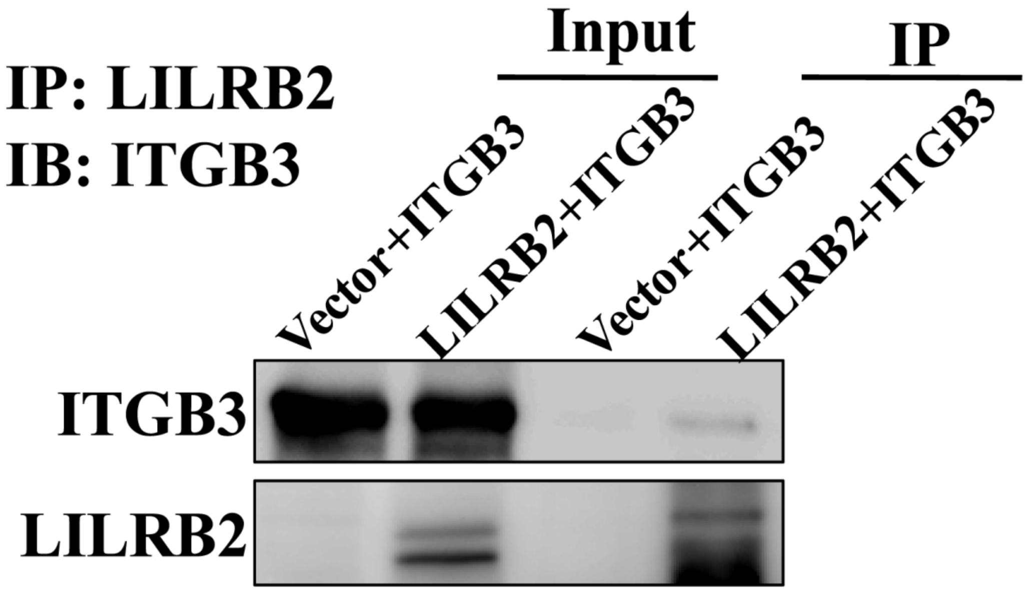

SFig 5: LILRB2 was directly associated with ITGB3. ITGB3 and LILRB2 (with an Fc tag) were cotransfected into 293T cells followed by pulldown with Protein A beads. ITGB3 and LILRB2 were further detected by western blotting.

STable 1: LILRB2/ANGPTL2 expression and clinical parameters

Variables	LILRB2 Expression			ANGPTL2 Expression		
	Low	High	p Value*	Low	High	p Value*
**Patients**	17	51		28	40	
**Age, yr**						
**<60**	6	22		13	15	
**≥60**	11	29	0.5693	15	25	0.4616
**Sex**						
**Male**	15	34		22	27	
**Female**	2	17	0.0861	6	13	0.3167
**Smoking index**						
**0**	7	27		12	22	
**≤400**	5	10	0.3390	8	7	0.2364
**>400**	5	14	0.6328	8	11	0.6237
**Histology**						
**Adenocarcinoma**	4	31		15	20	
**Squamous cell carcinoma**	13	20	0.0078	13	20	0.7718
**Pathologic stage**						
Ⅰ	6	16		11	10	
Ⅱ+Ⅲ	11	35	0.7647	17	30	0.2095
**TNM stage**						
**T1+T2**	12	37		21	28	
**T3+T4**	5	14	0.8760	7	12	0.6511

**Regional lymph node involvement**						
**N0**	6	21		8	19	
**N1-N3**	11	30	0.6677	20	21	0.1164

*, X^2 test
Abbreviations: TNM, tumor node metastasis

STable 2: Sequence for construction of LILRB2 shRNAs

Name	Sequence
**shRNA1-F**	TGATATGGCTGTCAGTATTATTCAAGAGATAATACTGACAGCCATATCTTTTTTC
**shRNA1-R**	TCGAGAAAAAAGATATGGCTGTCAGTATTATCTCTTGAATAATACTGACAGCCATATCA
**shRNA2-F**	TACTCCGTCTAAGATCAATATTCAAGAGATATTGATCTTAGACGGAGTTTTTTTC
**shRNA2-R**	TCGAGAAAAAAACTCCGTCTAAGATCAATATCTCTTGAATATTGATCTTAGACGGAGTA
**shRNA3-F**	TGCGGCTTCATTCTGTGTAATTCAAGAGATTACACAGAATGAAGCCGCTTTTTTC
**shRNA3-R**	TCGAGAAAAAAGCGGCTTCATTCTGTGTAATCTCTTGAATTACACAGAATGAAGCCGCA
**shRNA4-F**	TGCAGTTCCACACTTTCCTTTTCAAGAGAAAGGAAAGTGTGGAACTGCTTTTTTC
**shRNA4-R**	TCGAGAAAAAAGCAGTTCCACACTTTCCTTTCTCTTGAAAAGGAAAGTGTGGAACTGCA
**shRNA5-F**	TGGCATCTTGGATTACACGGATATTCAAGAGATATCCGTGTAATCCAAGATGCTTTTTTC
**shRNA5-R**	TCGAGAAAAAAGCATCTTGGATTACACGGATATCTCTTGAATATCCGTGTAATCCAAGATGCA

STable3: Multiplicative Quick Score systems

Stained malignant cells% (A)	Intensity (B)	Scores (AXB)
1 (0-4%)	0 (negative)	Low(≤2)
2 (5-19%)	1 (weak)	High(>2)
3 (20-39%)	2 (moderate)	
4 (40-59%)	3 (strong)	
5 (60-79%)		
6 (80-100%)		
